# Patellofemoral arthroplasty versus total knee arthroplasty for isolated patellofemoral osteoarthritis: a systematic review and meta-analysis

**DOI:** 10.1186/s13018-021-02414-5

**Published:** 2021-04-15

**Authors:** Guanrong Peng, Min Liu, Zhenhua Guan, Yunfei Hou, Qiang Liu, Xiaobo Sun, Xingyang Zhu, Wenjun Feng, Jianchun Zeng, Zhangrong Zhong, Yirong Zeng

**Affiliations:** 1 The First Clinical Medical School, Guangzhou University of Chinese Medicine, Jichang Road 12#, District Baiyun, Guangzhou, Guangdong China; 2 Yudu People’s Hospital, Huancheng North Road 2#, District Yudu, Ganzhou, Jiangxi China; 3 Peking University People’s Hospital, Arthritis Clinic and Research Center Beijing, Beijing, China; 4 Department of Orthopaedics, The First Affiliated Hospital of Guangzhou University of Chinese Medicine, Jichang Road 16#, District Baiyun, Guangzhou, Guangdong China

**Keywords:** Patellofemoral osteoarthritis, Patellofemoral arthroplasty, Total knee arthroplasty, Patient-reported outcome measure, Systematic review, Meta-analysis

## Abstract

**Background:**

Isolated patellofemoral osteoarthritis (PF-OA) is a common subtype of knee osteoarthritis, leading to a huge economic burden on health care systems. Although previous studies have shown that patellofemoral arthroplasty (PFA) and total knee arthroplasty (TKA) have good clinical effects, it remains largely unclear which treatment is more effective for patients with isolated PF-OA. We aimed to compare postoperative function, complications, revision rates, level of physical activity, and satisfaction rate between the two surgical techniques.

**Methods:**

Our study followed the Preferred Reporting Items for Systematic Reviews and Meta-Analysis (PRISMA) guidelines. Search of literature was conducted in MEDLINE, EMBASE, Cochrane Library, and Web of Science until November 2020. The included studies were those that provided direct comparison of postoperative outcomes between PFA and TKA. Data were extracted from eligible studies and combined to calculate the pooled odds ratio (OR) and 95% confidence interval (CI). Sensitivity analysis and subgroup analysis were conducted to evaluate heterogeneity between the two groups.

**Results:**

A total of 7 eligible studies (3 recent randomized controlled trials and 4 nonrandomized controlled trials) were included in this meta-analysis. The pooled results showed that both the PFA group and the TKA group had improved postoperative indicators, suggesting that the two operation modes could improve the knee function and quality of life of patients. Throughout the first 2 years postoperatively, higher activity level, and better functional recovery were observed for PFA compared with TKA in this study; moreover, the differences between the two operation modes were statistically significant (*p* < 0.05). We found no significant difference in complications, revision rates, and satisfaction rate between the two procedures.

**Conclusion:**

Although there was no observed difference in the complications, revision rates, and satisfaction rate between PFA and TKA, PFA was superior to TKA in terms of knee function and physical activity in the first 2 years postoperatively. Therefore, PFA is a safe, effective, and less invasive treatment for patients with isolated PF-OA. Our findings are consistent with the systematic review of current evidence that PFA may be more suitable for younger patients with high activity needs. Patient selection is, therefore, thought to be of paramount importance. Individualized surgical plan should be designed according to the patient’s age, BMI, KOA site, and activity level and combined with the doctor’s personal experience.

**Supplementary Information:**

The online version contains supplementary material available at 10.1186/s13018-021-02414-5.

## Introduction

Osteoarthritis (OA) of the knee is a common form of degenerative joint disease, which affects individuals all over the world. Over 100,000 primary knee arthroplasties are conducted annually in the United Kingdom (UK) and were predicted to increase 6-fold by 2030 (UK National Joint Registry 2014) [1]. One-third of OA patients are affected in only one compartment [2]. Studies have shown that the isolated patellofemoral osteoarthritis (PF-OA) affects 11% to 24% of the general population with knee pain who are 55 years and older, with a female preponderance [3, 4]. PF-OA has a substantial negative socioeconomic impact on the health care system due to a high prevalence and the chronic relapsing nature of symptoms.

In the early stage of isolated PF-OA, the treatment options include conservative therapies and minor surgical procedures, such as arthroscopy, cartilage stimulation with microfractures, lateral retinacular release, tibial tuberosity transposition, and cartilage replacement procedures [5–11]. In the late stages, when severe pain is present, joint arthroplasty such as patellofemoral arthroplasty (PFA) and total knee arthroplasty (TKA) may be recommended [12–14]. TKA is perceived to be the gold standard treatment of symptomatic late-stage knee osteoarthritis (KOA) and provides good long-term outcomes [15]. However, for the isolated PF-OA, TKA is not the preferred treatment strategy, especially for younger and highly active patients. PFA is an alternative to TKA for the treatment of isolated PF-OA [16, 17], preserving both tibiofemoral joints and ligamentous structures as a less invasive operation, and enabling a faster recovery [18]. A previous review suggested PFA and TKA have similar results in terms of complications and reoperation rates for isolated PF-OA [19]. However, a recent randomized controlled trial (RCT) comparing TKA with the Avon PFA in patients with isolated patellofemoral disease found a greater overall knee-specific quality of life and improved range of movement 2 years postoperatively for the Avon group [20]. PFA and TKA are two kinds of prostheses with different designs and require different surgical techniques. Choosing the appropriate prosthesis type for isolated PF-OA surgery remains somewhat controversial for patients with isolated PF-OA. Therefore, we conducted a systematic review and meta-analysis to compare PFA and TKA for patients with isolated PF-OA.

The aim of our study was to quantitatively evaluate if the outcomes of PFA and TKA differs with regard to (1) postoperative complications, (2) rates of revision, (3) function, (4) postoperative physical activity, and (5) satisfaction rate.

## Methods

### Search strategy

We strictly conducted this study in accordance with the PRISMA statement (Preferred Reporting Items for Systematic Reviews and Meta-Analyses) [21]. First, the research protocol for this review was determined by all coauthors and then the literature searches begin. With the assistance of an experienced librarian, two authors (Guanrong Peng and Min Liu) developed the search strategy following the PICOS methodology. By November 13, 2020, according to the comprehensive retrieval strategy, we have systematically searched the four major electronic databases, including MEDLINE (through PubMed), EMBASE (through OvidSP), SCI (through Web of Science), and CENTRAL (Cochrane Central Register of Controlled Trials, through the Cochrane Library). To maximize sensitivity, we had no restrictions on the language and publication date of the articles in the whole retrieval process. The literature search strategy for the four databases followed Medical Subject Headings combination with terms. Additionally, the reference lists of each comparative study and reviews were also examined to identify additional relevant studies. The detailed search strategy of this study is shown in Additional file 1.

### Inclusion and exclusion criteria

Two authors (Guanrong Peng and Min Liu) independently evaluated the search results by scanning the titles or abstracts or full text. Eligible studies were included in the current systematic review. If there was a disagreement between the two authors on qualification issues, a consensus could be reached by consultation with another author (Yirong Zeng).

All RCTs or non-randomized controlled trials (nRCTs) that directly compared PFA with TKA to treat isolated PF-OA were identified and included. The inclusion criteria were (1) in the original comparative studies, all surgical procedures were primary PFA and TKA; (2) there was no difference in baseline data between the two groups, including age, gender, body mass index (BMI), previous procedures and preoperative outcome measures; (3) complete data were available to calculate the pooled odds ratios (OR) with 95% confidence interval (CI); and (4) at least one of the following outcome indicators was reported: function (e.g., knee function score or range of motion), daily physical activity score, complications (e.g., pain, postoperative joint stiffness, infection, thrombosis, periprosthetic fractures), revision rate of prostheses, and postoperative satisfaction. Exclusion criteria were as follows: (1) literature without available data due to incomplete or unclear information; (2) revision surgery was included; (3) included other surgical procedures; (4) repetitive articles, unable to obtain full text, conference and case reports, reviews, systematic reviews and expert opinions; (5) animal experiments; (6) authors could not distinguish languages; (7) non-conformity with inclusion criteria; and (8) without clear outcome measures and evaluation standard of curative effect.

### Study quality assessment

To determine whether biases might have affected the results, the Cochrane Risk Bias Tool (CRBT) was used to assess the risk of bias in RCTs [22]. We assessed the risk of bias in the nonrandomized studies using the Risk of Bias in Non-Randomized Studies of Interventions (ROBINS-I) assessment tool [23]. The ROBINS-I tool evaluates bias including the following aspects: bias due to confounding, bias in the selection of participants, bias in measurement of interventions, bias due to departures from intended interventions, bias due to missing key data, bias in measurement of outcomes, and bias in selection of the reported result. Each study included was independently assessed by two authors (Guanrong Peng and Min Liu). In the case of any controversy, a final consensus was reached through discussion or resolved by a third author (Yirong Zeng).

### Data extraction

The first author extracted data from all included studies based on a standardized date collection form, and then two other authors repeated this process to extract data. This standardized data extraction format included the following aspects: (1) study general information (i.e., author, country, journal, year of publication and type of study); (2) population information of study (i.e., sex, age, BMI, and diagnosis); (3) surgery type and follow-up time; and (4) outcome measures (i.e., functional outcomes of knee, daily physical activity score, complications, revision rates, health-related quality of life (HRQOL), and satisfaction rate). Complications were defined as postoperative unfriendly issues. Except the study by Ivan Kamikovski et al. [24], we contacted the author by phone, email, or other means for more information when the key data of the studies were missed.

### Statistical analysis

We used the OR and associated 95% CI for statistical analysis of each study containing dichotomous variables, such as complications, revision incidence, and satisfaction rate. For continuous variables, including daily physical activity score and knee function score, the mean difference (MD) or standard mean difference (SMD) was used. For continuous data with mean and range values as the outcomes, we used statistical algorithms to calculate standard deviations [24]. Our system analysis only counted those studies that gave both means and standard deviations. The heterogeneity between studies was evaluated by *I*^2^ and *P* value. While the statistical result was *P* > 0.1 or *I*^2^ < 50% (no heterogeneity among the studies), the fixed effect model was applied to merge the effect quantities. Otherwise, the random effect model was used.

After selecting the test mode, sensitivity analysis was conducted when necessary to evaluate the stability of the results. We obtained more specific conclusions through subgroup analysis if data were available. Moreover, forest plots were used to explain the results of individual studies and estimated the effect of each merger separately. Funnel plots were used to detect publication bias for any results. In this system analysis, Review Manager (version 5.3.5 for Windows, the Cochrane Collaboration, the Nordic Cochrane Centre, Copenhagen, 2014) was used for all statistical analyses.

## Results

### Study selection

**Fig. 1:**
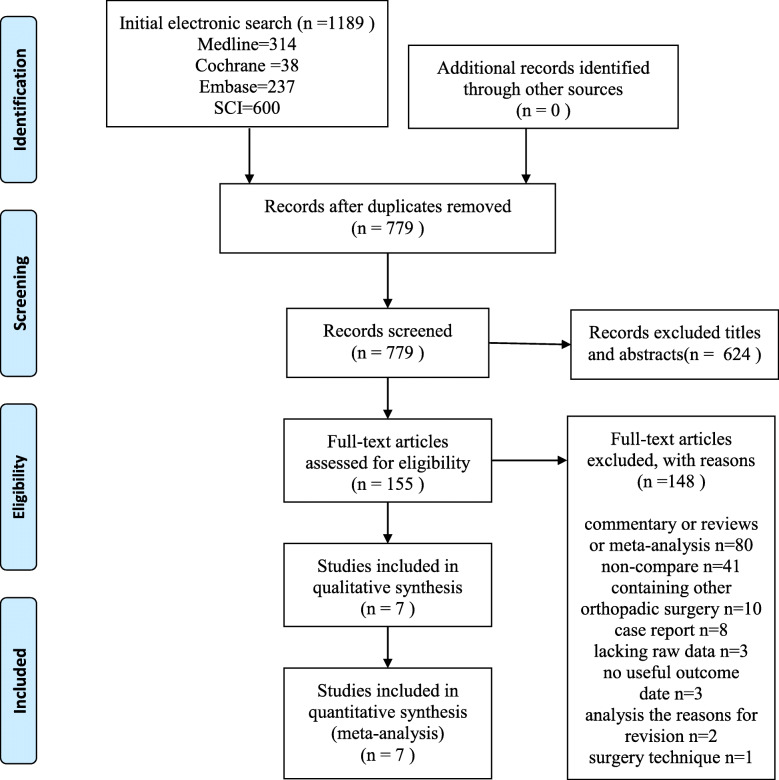
Flow diagram for search strategy

We extracted a total of 1189 potentially relevant citations from the four electronic databases (MEDLINE = 314, EMBASE = 237, SCI = 600, CENTRAL = 38). First, 410 repetitive studies were eliminated by citation management software. Second, 624 irrelevant citations were excluded by browsing the titles and abstracts. Third, 148 of which were excluded again for reasons, such as commentary (or reviews, *n* = 80), no control groups (*n* = 41), containing other orthopedic surgery (*n* = 10), case report (*n* = 8), lacking raw data (*n* = 3), no useful outcome date (*n* = 3), analysis of reasons for revision (*n* = 2), and surgery technique (*n* = 1). Finally, a total of 7 articles [15, 20, 25–29] were included in this research. The detailed screening process of this study was shown in Fig. 1.

### Study characteristics and quality

In these 7 studies that included 505 patients (509 knees) with isolated PF-OA, there were 246 cases (250 knees) in the PFA group and 259 patients (259 knees) in the TKA group. Two of the seven studies were from Denmark [15, 20], two from the UK [25, 27], one from Canada [28], one from Germany [29], and one from the USA [26]. Two studies were published in 2020 [15, 27], two in 2019 [25, 28], two in 2018 [20, 29], and one in 2010 [26]. The follow-up period of the seven studies was differ ranging from 1 year [27] to 15 [25] years. The mean age ranged from 50 years [28] to 72 years [29]. Study characteristics, information of related research journals, interventions, patient demographic details, and clinical results for the 7 studies in this meta-analysis were shown in Tables 1 and 2.

**Table 1:** Characteristics of the studies included in the review

Author	Year	Country	Journal	Design	No (patient)	Group	NO (patient)	Age (Year)	Gender (M/F)	BMI	Diagnosis	Model
Fredborg et al. [15]	2020	Denmark	*Bone & Joint Journal*	RCT	100	PFA	50	64.0 ±8.6	11/39	28.0 ±4.7	IPFOA	Stryker
						TKA	50	64.4 ±9.3	12/38	27.8 ±4.1		DePuy
Joseph et al. [27]	2020	UK	*Bone & Joint Journal*	RCT	60	PFA	31	64.7 ±10.5	9/22	28.9 ±6.7	IPFOA	Zimmer Biomet Stryker
						TKA	29	64.4 ±12.8	3/26	29.2 ±4.2		
Odgaard et al. [20]	2018	Denmark	*Clinical Orthopaedics and Related Research*	RCT	100	PFA	50	64.0±8.9	23/77	NC	IPFOA	Stryker
						TKA	50					DePuy
Clement et al. [25]	2019	UK	*Bone & Joint Journal*	retrospective	108	PFA	54	62.4 ±11.3	5/49	NC	IPFOA	NC
						TKA	54	64.0 ±10.8	8/46			Stryker
Kamikovski et al. [28]	2019	Canada	*Journal of Arthroplasty*	prospective	42	PFA	19 (23knees)	50.4 ±3.4	16/3	28.5 ±5.6	IPFOA	Stryker, Zimmer Biomet
						TKA	23	50.5 ±2.4	19/4	28.2 ±5.8		Zimmer Biomet
Perrone et al. [29]	2018	Germany	*Technology and Health Care*	retrospective	50	PFA	19	52.4 ±10.6	6/13	27.9 ±4.4	IPFOA	Zimmer Biomet
						TKA	31	71.9 ±9.8	12/19	29.4 ±4.5	KOA	Zimmer Biomet
Dahm et al. [26]	2010	USA	*American Journal of Orthopedics*	retrospective	45	PFA	23	60(39-81)	NC	30	IPFOA	Stryker
						TKA	22	69(44-83)		30		Zimmer ,DePuy

*RCT* randomized controlled trial, *No* the number of patients, *PFA* patellofemoral arthroplasty, *TKA* total knee arthroplasty, *NC* not clear, *M/F* male/female, *BMI* body mass index, *IPFOA* isolated patellofemoral osteoarthritis, *KOA* knee osteoarthritis, *USA* United States of America, *UK* United Kingdom

**Table 2:** Summary of clinic outcomes (throughout the first 2 years after operation) for each study

Author	Year	Group	Follow-up	Complication (revision)	Function score	UCLA activity score	HRQOL	Satisfaction (YES/NO)
Fredborg et al. [15]	2020	PFA	2 Y	8 (1)	NC	NC	0.84 ± 0.13 (EQ-5D)	NC
		TKA		13 (0)			0.79 ± 0.20 (EQ-5D)	
Joseph et al. [27]	2020	PFA	1–5 Y	4 (0)	13.7 ± 8.3 (OKS)	5.5±1.6	0.68 ± 0.24 (EQ-5D)	17/3
		TKA		9 (0)	15.4 ± 9.2 (OKS)	4.7±1.2	0.63 ± 0.34 (EQ-5D)	21/2
Odgaard et al. [20]	2018	PFA	2 Y	4 (2)	8.0 ± 7.4 (OKS)	NC	10.6 ± 3.4 (KOOS QOL)	NC
		TKA		7 (0)	13.0 ± 7.4 (OKS)		9.6 ± 3.4 (KOOS QOL)	
Clement et al. [25]	2019	PFA	10.1 (8–15) Y	8 (4)	31.0 ± 10.4 (OKS)	NC	47.8 ± 11.0 (SF-12)	39/6
		TKA	9.7 (8 - 12) Y	10 (1)	31.2 ± 10.5 (OKS)		48.6±10.8 (SF-12)	36/10
Kamikovski et al. [28]	2019	PFA	5.16 ±1.52 Y	NC	53.7 ± 12.2 (WOMAC)	5.7± 1.4	4.2 ± 4.3 (KOOS QOL)	NC
		TKA	5.38 ± 1.25 Y		59.2 ± 9.0 (WOMAC)	6.7 ±1.7	10.2 ±3.2 (KOOSQOL)	
Perrone et al. [29]	2018	PFA	32.7 ± 9.3 M	5 (1)	29.5 ± 10.5 (OKS)	NC	NC	NC
		TKA		6 (3)	38.7 ±8.4 (OKS)			
Dahm et al. [26]	2010	PFA	29 (24–49) M	6 (0)	84±49.7 (AKSS)	6.6±4.6	NC	17/6
		TKA	27 (24–33) M	6 (0)	73±39.5 (AKSS)	4.2±3.4		18/4

*OKS* Oxford Knee Score, OKS is a 12-item knee function assessment, ranging from 0 (best score) to 48 (worst score); *WOMAC* Western Ontario and McMaster Universities Osteoarthritis Index, WOMAC function score, primary outcome measure, range ranging from 0 (worst score) to 68 (best score); *UCLA* University of California Los Angeles, UCLA activity score ranging from 0 (worst score) to 10 (best score); *AKSS* American Knee Society scoring, AKSS measures function, range from 0 (worst outcome) to 100 (best outcome); *KOOSQOL* The Knee injury and Osteoarthritis Outcome Score quality of life, ranging from 0 (worst score) to 16 (best score); *Y* year; *M* month; *PFA* patellofemoral arthroplasty; *TKA* total knee arthroplasty; *HRQOL* health-related quality of life; *SF-12* the 12-Item Short-Form Health Survey; *EQ-5D* EuroQol five-dimension questionnaire

We included 3 RCTs [15, 20, 27] and 4 nRCTs [25, 26, 28, 29]. Subgroup analysis was used in the 3 RCTs. RCTs were assessed using the CRBT according to 6 domains of potential biases, while nRCTs were evaluated using ROBINS-I according to 7 domains of potential biases. The specific test qualities of all studies were shown in Additional files 2 and 3.

### Complications

**Fig. 2:**
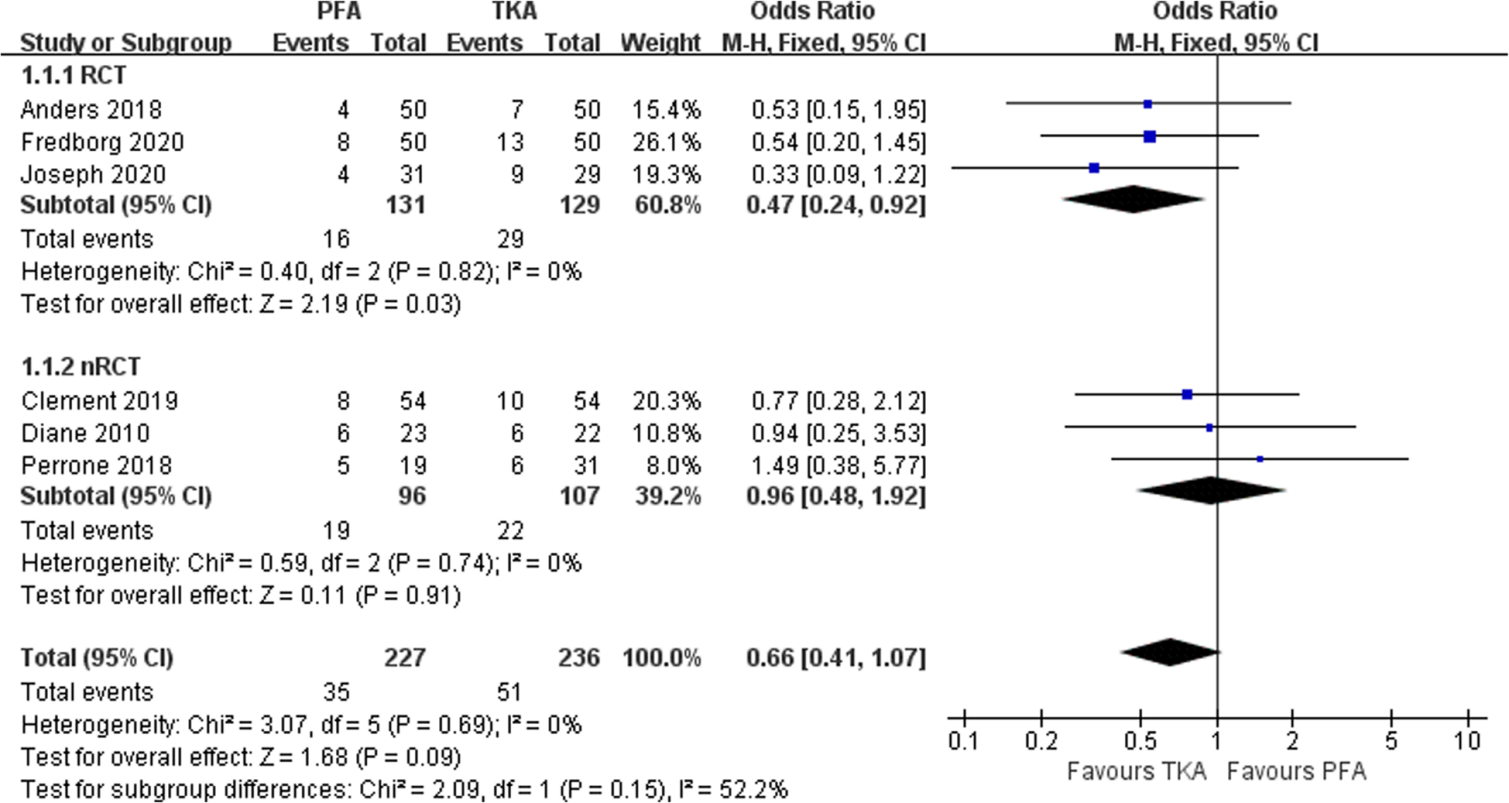
Comparison of complications incidence between PFA and TKA groups

**Fig. 3:**
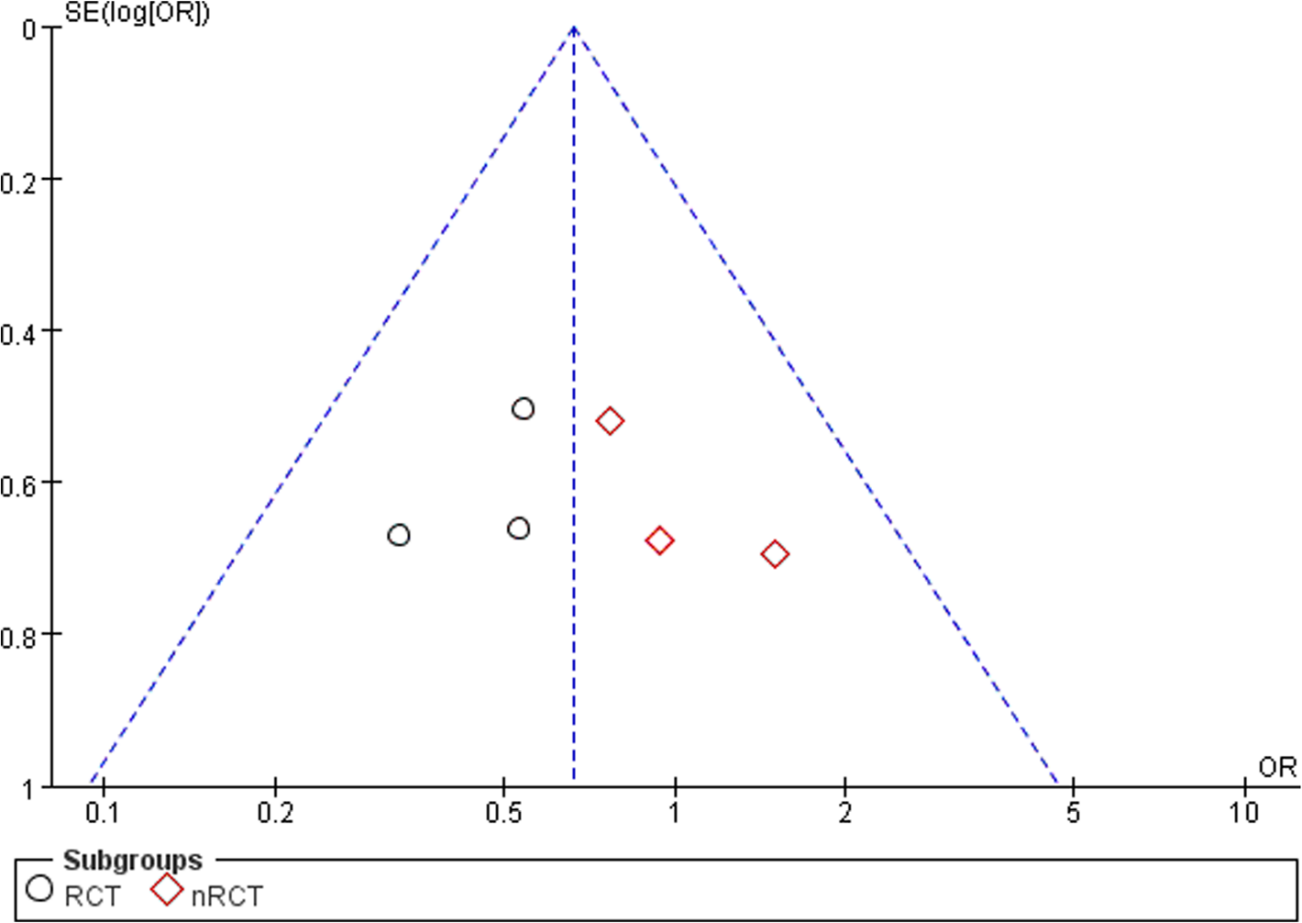
Funnel plot illustrating a meta-analysis of the incidence of complications between PFA and TKA. OR, odds ratio; SE, standard error

Complications were defined as postoperative unfriendly issues. Except the study by Ivan Kamikovski et al. [28], a total of 463 patients were evaluated in 6 studies [15, 20, 25–27, 29] to estimate the incidence of complications. Main complications included pain, joint stiffness, activity limitation, infection, unequal length of lower limbs, deep vein thrombosis, revision, operation area paralysis, and periprosthetic fracture. We divided the studies into the RCT and nRCT groups for subgroup analysis. Six articles were tested for heterogeneity, which showed that the heterogeneity of the selected studies was not statistically significant(*P* = 0.69> 0.1, *I*^2^ = 0% < 50%; Fig. 2). The pooled data showed no statistical significance in the incidence of complications between the two groups in the fixed effect model (OR 0.66, 95% CI 0.41–1.07, *Z* = 1.68, *P* = 0.09; Fig. 2). The funnel symmetry demonstrated that there was no significant publication bias regarding the incidence of complications between the two group (Fig. 3). In other words, there was no significant difference in the incidence of complications between the two groups.

### Revision rates

**Fig. 4:**
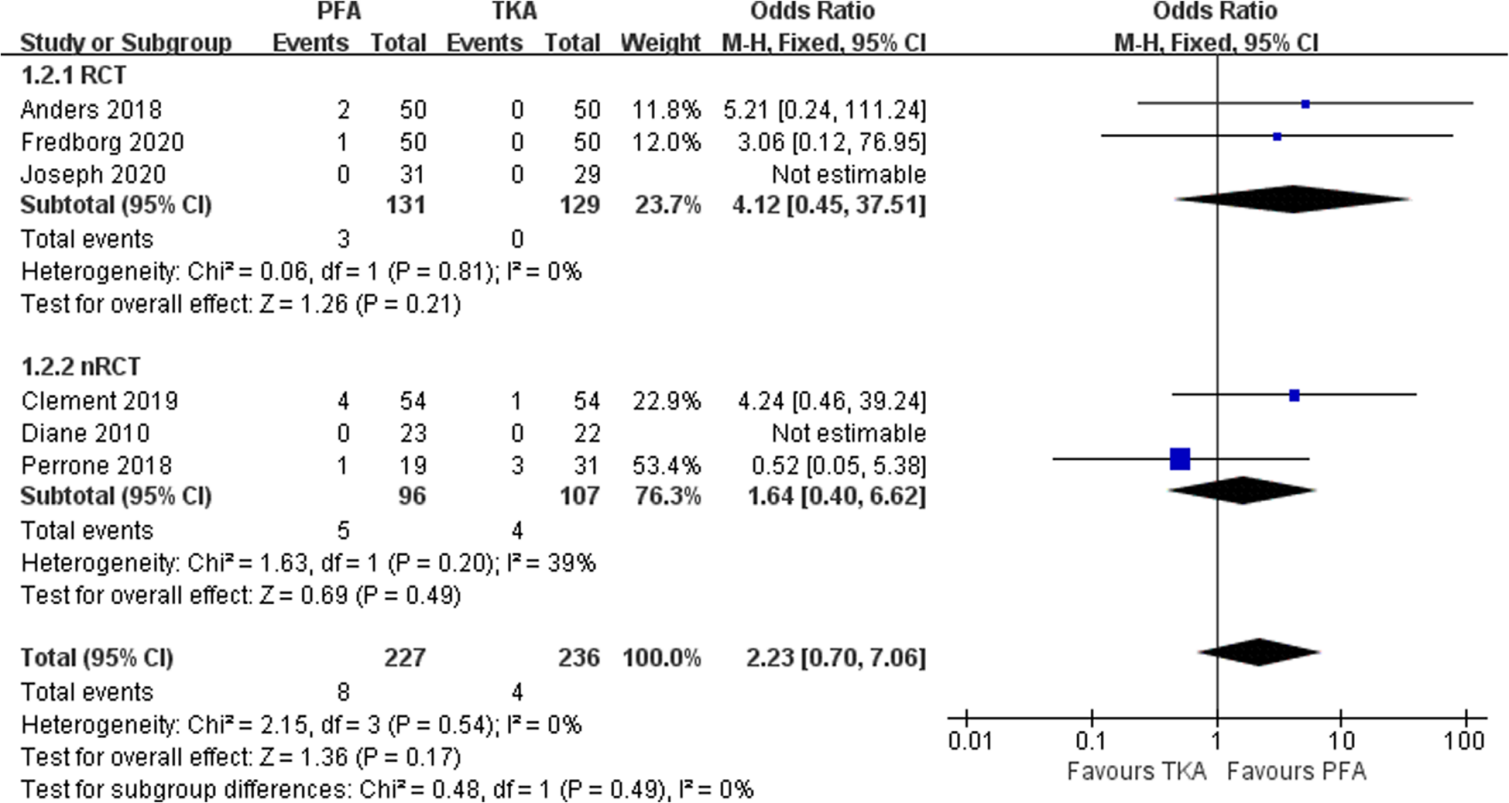
Comparison of the revision rates between PFA and TKA groups

Six studies assessed the incidence of revisions in a total of 463 patients [15, 20, 25–27, 29]. Revision surgery was reported in both groups. However, subgroup analysis indicated that the revision rates between the PFA and TKA groups were not statistically different (OR 2.23, 95% CI 0.70–7.06, *Z* = 1.36, *P* = 0.17; Fig. 4). That was to say, the revision rate of PFA was not higher than that of TKA.

### Functional results

**Fig. 5:**
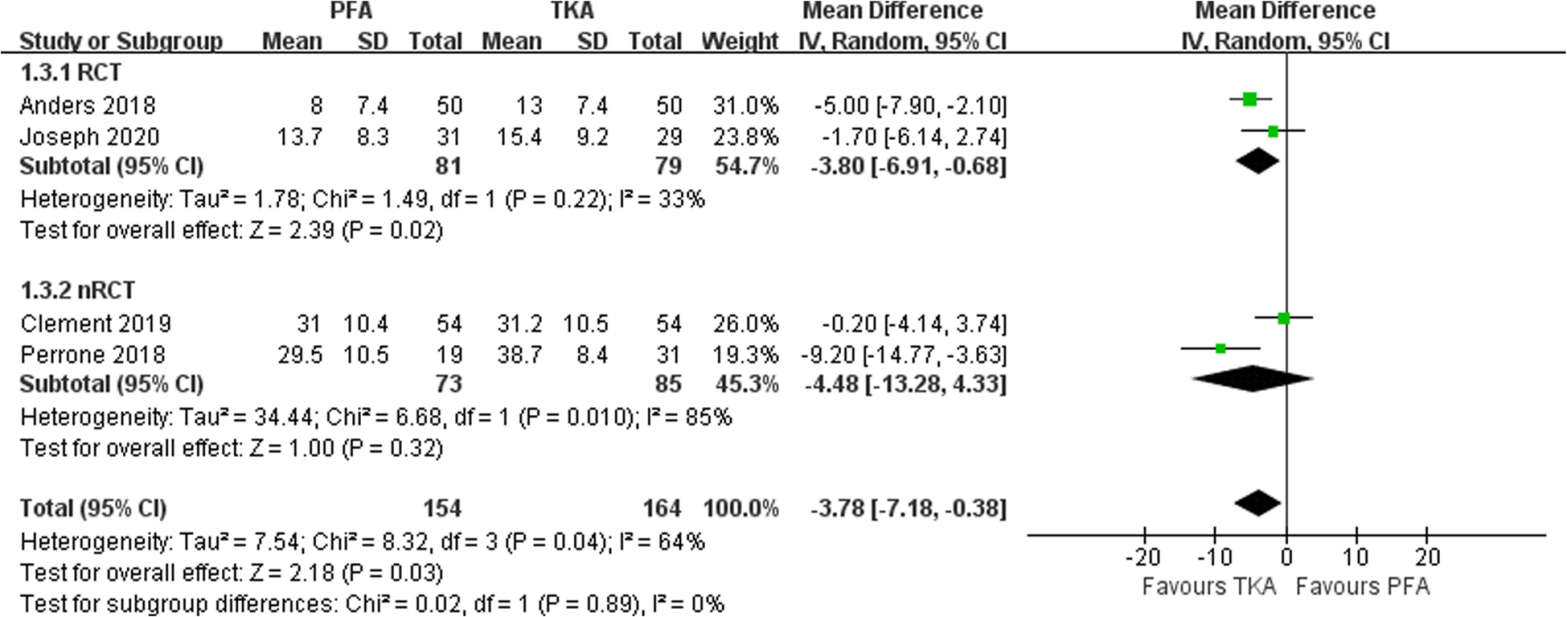
Forest plot of RCTs and nRCT reporting function results (throughout the first 2 years after operation) for PFA and TKA patients

The results of knee function were compared by the patient-reported outcome measures. Fredborg et al. [15] did not give a knee function score. Therefore, six [20, 25–29] out of the seven studies assessed knee function using different knee function scoring systems. Ivan Kamikovski et al. [28] used Western Ontario and McMaster Universities Osteoarthritis Index (WOMAC) functional score (0 best, 68 worst). Dahm et al. [26] used the American Knee Society scoring system (AKSS, 0 worst, 100 best). The remaining four studies used the Oxford Knee Score system (OKS, 0 worst, 48 best) [20, 25, 27, 29]. Five out of six studies reported better functional scores in the PFA group [20, 25 - 27, 29]. Furthermore, among four studies that used the OKS [20, 25, 27, 29], the pooled data showed that there was a statistical difference between PFA and TKA (MD − 3.78; 95% CI, − 7.18, − 0.38; *Z* = 2.18, *P =* 0.03 < 0.05; Fig. 5).

### Daily physical activity scores

**Fig. 6:**
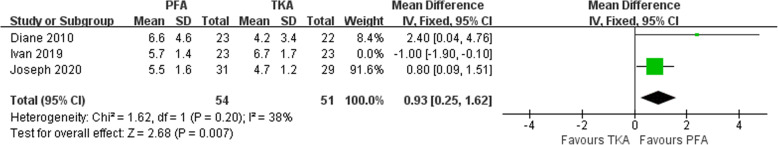
Comparison of the Physical Activity Scores between PFA and TKA groups

Three studies reported using the University of California Los Angeles Physical Activity Rating Scale (UCLA, 0 worst, 10 best) to assess physical activity [26–28]. There was significant heterogeneity among these three studies(*P* = 0.002< 0.1, *I*^2^ = 84% > 50%). The study of Ivan Kamikovski et al. [28] was excluded in sensitivity analysis. While the UCLA activity scores of the two groups were improved in the final follow-up, the recovery of physical activity was better in the PFA group (MD 0.93, 95% CI 0.25–1.62, *Z* = 2.68, *P* = 0.007; Fig. 6).

### Satisfaction rate

**Fig. 7:**
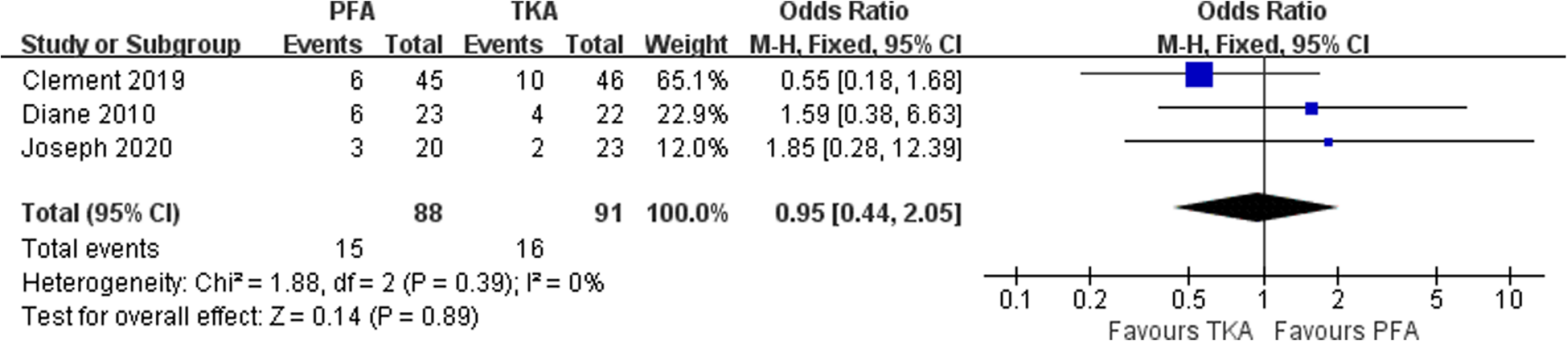
Forest plot of reporting satisfaction rates for PFA and TKA patients

A total of 213 patients were finally followed up for the rate of satisfaction in three studies [25–27]. Compared with TKA, PFA had higher functional results and physical activity scores. However, the pooled data showed no significant difference in satisfaction rates between PFA and TKA (OR 0.95, 95% CI 0.44–2.05, *Z* = 0.14, *P* = 0.89; Fig. 7).

## Discussion

In recent years, studies have shown that both PFA and TKA have achieved satisfactory results in the treatment of patients with PF-OA [30–35]. However, surgical treatment options for severe symptomatic isolated PF-OA remain controversial. We objectively and quantitatively designed this meta-analysis to compare the effectiveness of PFA and TKA for isolated PF-OA. Perrone et al. [29] noted that patient-reported outcome measures (PROMs) are extensively used as an important outcomes measure to evaluate patients undergoing joint replacement surgery. Studies included in our meta-analysis also performed PROMs as evaluation methods of outcome. All the outcome measures were improved in both PFA and TKA groups, suggesting that the two operation modes could improve the function and quality of life. Throughout the first 2 years after operation, a higher physical activity level and better functional recovery were observed for PFA compared with TKA, which was consistent with the conclusion of previous studies [15, 20, 28]. The pooled data showed no statistical difference between the two groups in terms of complications, revision rates, and satisfaction rate.

The premise for any surgical operation to obtain satisfactory clinical efficacy and high safety is to strictly grasp its indications and contraindications. Therefore, selecting the suitable patients for PFA is a challenge for the surgeons. PFA has been defined by 6 classic indications and 11 consensus contraindications [36–38]. Indications include (1) isolated degenerative osteoarthritis of patellofemoral joint, (2) severe patellofemoral joint degeneration with ineffective long-term conservative treatment (at least 3–6 months) and/or failure of conservative surgery, (3) posttraumatic patellofemoral arthritis, (4) generalized grade III patellofemoral arthritis chondropathy, (5) failure of a previous extensor unloading surgical procedure, and (6) degenerative changes with or without instability due to patellofemoral dysplasia. The contraindications include (1) no conservative treatment or other sources of pain cannot be excluded, (2) arthritis of greater than Kellgren-Lawrence Grade 1 involving the tibiofemoral articulation, (3) osteoarthritis or chondrosis of the patellofemoral joint of Grade 3 or less, (4) uncorrected patellofemoral instability or malalignment, (5) patella baja, (6) systemic inflammatory arthritis, (7) active infection, (8) uncorrected tibiofemoral mechanical malalignment (varus> 5° or valgus> 8°), (9) psychogenic pain, (10) evidence of chronic regional pain syndrome, and (11) fixed loss of knee range of motion (− 10° of extension to 110° of flexion at a minimum).

PFA is a relatively early clinical technology, so far a large number of scholars have carried out research and reported their results. Leadbetter WB et al. [37] reported that factors including gender, age(< 40 years), obesity (BMI of > 30 kg/m^2^), primary osteoarthritis, patella alta, and a high activity level might compromise the clinical outcomes of PFA. Van Jonbergen et al. [39] considered that obesity (BMI > 30) was not only a risk factor for patellofemoral arthritis, but also a risk factor for revision after PFA. Nevertheless, Jared et al. [40] proffered that 35 obese (BMI > 30) patients with isolated PF-OA could achieve the same improvement in function as non-obese (BMI: 18.5–25) patients following PFA, and there was no difference in PFA revision rate between the two. As is known to all, the mold of implant is one of the most important considerations affecting the clinical results. Modern PFA implants have been widely used due to their higher functional success rates and lower complication rate. Moreover, as technology advances, including custom implants and robotic assistive programs, the advantages of PFA may be further enhanced [41, 42]. Additionally, studies have already shown that 66% to 100% of patients with PFA achieved good to excellent results over a 3- to 17-year follow-up [43–47]. For example, Cartier et al. [44] reported on a study of the first-generation of PFA that 55 of 65 patients achieved good to excellent results at 4 years. Van der List JP et al. [47] found that the 5-, 10-, 15- and 20-year survival rate of PFA prosthesis was 91.7%, 83.3%, 74.9%, and 66.6%, respectively. In addition, in a study of 62 patients with a mean follow-up of 5.0 ± 2.1 years, Jonas Pogorzelski et al. [46] demonstrated that 94% of the patients with PFA were able to return to the same or higher level of exercise, while 74% of the patients showed improved ability to perform sports.

Admittedly, the “gold standard” for the primary treatment of symptomatic advanced KOA is TKA. Meanwhile, TKA is considered as a benchmark for the treatment of isolated PF-OA [15]. Nevertheless, the resection of cruciate ligament in TKA affects the participation and range of motion in high demand activities [48, 49]. Besides, the revision rate increased by two to three times among young patients who received TKA in the following decades [50]. In recent years, many scholars have shown renewed interest in the field of PFA. With the improvement of PFA prosthesis design and surgical techniques, modern PFA has become a reasonable choice for young isolated PF-OA patients to delay TKA [44]. It has shown a three to four times improved survivorship than the first generation implants [51]. There were 3 RCTs [15, 20, 27] and 4 nRCTs [25, 26, 28, 29] comparing PFA and TKA for patients with isolated PF-OA found no difference between the interventions in terms of satisfaction rates, complications, and revision rates. Shubin Stein et al. [52] noted that the majority of PFA patients could return to preoperative level of physical activity, which was also found in our study. Leadbetter et al. [53] discovered that PFA patients could return to tennis, ballet, skiing and other activities in the short term.

Compared with TKA, the advantages of PFA include lower intraoperative blood loss, shorter surgical duration and tourniquet time, the minimal femoral bone loss as well as the preservation of the tibiofemoral articulation, menisci, and ligaments [41, 42]. The minimally invasive PFA technology allows a faster rehabilitation and better range of motion and function for young patients in a short time after operation. Notably, for younger patients with isolated PF-OA, TKA is defined as a more invasive procedure because it requires the replacement of two healthy tibiofemoral joints [31, 37]. Compared with the older population, TKA has a higher revision rate among younger patients [54]. What is more, PFA is at least 1.0% more effective than TKA in the terms of cost-effectiveness [50]. PFA may bring good clinical effects and economic benefits to patients when it is accepted as a practical technique for the treatment of isolated PF-OA. Additionally, if tibiofemoral arthritis progression or implant failure occurs, PFA can be used as a bridge operation for TKA in the future potentially reducing the revision rates. Of note, based on a proper patient selection, precise prosthesis design and accurate surgical technique, Shubin Stein et al. [52] believed that PFA should be more and more popular in the young active patients with isolated PF-OA. However, previous studies have been reported that the progression of tibiofemoral arthritis is considered to be a common cause of failure of modern PFA prostheses [31, 44]. Furthermore, Dahm et al. suggested that patients with idiopathic PF-OA may be more likely to develop generalized tibiofemoral arthritis [26]. Odgaard et al. [20] reported that there were two revisions within 2 years in patients with PFA. Selecting the right patient for PFA, therefore, thought to be of paramount importance.

To the best of our knowledge, this appears to be the first meta-analysis to compare PFA and TKA for patients with isolated PF-OA in terms of postoperative function, complications, revision rate, physical activity and satisfaction. Furthermore, the great strength of this study is that it's the latest meta-analysis to objectively and quantitatively compare the efficacy between the two surgical techniques. Compared with a previous systematic review and meta-analysis [51], we included a number of new clinical studies [15, 20, 25, 27–29] up to 2020. We noted that an excellent meta-analysis has been published previously by Dy et al. [51], but the purpose was mainly to compare the postoperative complications of PFA and TKA. Consequently, our results are more up-to-date. Furthermore, we conducted the current study in accordance with the PRISMA statement and critically evaluated the quality of all selected studies.

However, our meta-analysis has some limitations, which should be considered when interpreting the results. First, some studies have been inevitably omitted or not identified due to search strategy, although we consulted a professional librarian and optimizing the retrieval strategy. Second, only 3 of 7 studies were RCTs in our research, in which it is very hard to blind participants and investigators. Because surgical procedures are determined by the patient and the physician, it is difficult to maintain a baseline balance between the PFA and TKA groups. Third, the results of our systematic analysis may be influenced by different prosthesis types, surgical technique and postoperative care. Fourth, the follow-up period of 7 studies included in this meta-analysis is heterogenous ranging from 1 to 15 years, which may have introduced recall bias and varying time points for collection of postoperative patient-reported outcome measures. Finally, this meta-analysis included studies that chose results cutoff of two years after surgery, the postoperative outcomes may also be associated with the follow-up time. Therefore, longer follow-up is necessary to compare the medium and long-term efficacy of the two surgical methods.

## Conclusions

In this up-to-date meta-analysis, while satisfactory clinical effectiveness was achieved by both PFA and TKA, PFA showed superior functional results and UCLA scores compared with TKA. There were no significant differences in complications, revision rates and satisfaction rate between PFA and TKA for isolated PF-OA. Thus, younger active patients may be good candidates for PFA. High-quality, multicenter, large sample prospective randomized controlled trials are needed to confirm these findings. In general, our study may provide more reliable objective evidence for clinical treatment of isolated PF-OA.

## Supplementary Information

**Additional file 1.** Search strategy.

**Additional file 2.** Methodological assessment according to seven domains of potential biases (ROBINS-I).

**Additional file 3.** Methodological assessment according to six domains of potential biases(Cochrane Risk of Bias Tool).

## Data Availability

The datasets used and/or analyzed during this study are not publicly available due to feasibility, but are available from the corresponding authors on reasonable request.

## Abbreviations

OA: Osteoarthritis
KOA: Knee osteoarthritis
UK: United Kingdom
PF-OA: Patellofemoral osteoarthritis
TKA: Total knee arthroplasty
PFA: Patellofemoral arthroplasty
RCT: Randomized controlled trial
nRCT: Nonrandomized controlled trials
BMI: Body mass index
OR: Odds ratios
CI: Confidence interval
MD: Mean difference
UCLA: University of California Los Angeles
