# Supplementary material for: Patellofemoral arthroplasty versus total knee arthroplasty for isolated patellofemoral osteoarthritis: a systematic review and meta-analysis
Source: J Orthop Surg Res. 2021 Apr 15;16:264. doi: 10.1186/s13018-021-02414-5 (PMC8048312; doi:10.1186/s13018-021-02414-5)
Supplement: Supplementary file 1 — Additional file 1. Search strategy. [file 13018_2021_2414_MOESM1_ESM.docx]

**Appendix 1 Search Strategy**

**Pubmed**

**#1**"Patellofemoral Joint"[Mesh]

**#2**

((((((((((Joint, Patellofemoral[Title/Abstract]) OR (Joints, Patellofemoral[Title/Abstract])) OR (Patellofemoral Joints[Title/Abstract])) OR (Patellofemoral Articulation[Title/Abstract])) OR (Articulation, Patellofemoral[Title/Abstract])) OR (Articulations, Patellofemoral[Title/Abstract])) OR (Patellofemoral Articulations[Title/Abstract])) OR (Femoropatellar Articulation[Title/Abstract])) OR (Articulation, Femoropatellar[Title/Abstract])) OR (Articulations, Femoropatellar[Title/Abstract])) OR (Femoropatellar Articulations[Title/Abstract])

**#3 #1 OR #2**

**#4:** "Osteoarthritis"[Mesh]

**#5**

(((((((((Osteoarthritides[Title/Abstract]) OR (Osteoarthrosis[Title/Abstract])) OR (Osteoarthroses[Title/Abstract])) OR (Arthritis, Degenerative[Title/Abstract])) OR (Arthritides, Degenerative[Title/Abstract])) OR (Degenerative Arthritides[Title/Abstract])) OR (Degenerative Arthritis[Title/Abstract])) OR (Arthrosis[Title/Abstract])) OR (Arthroses[Title/Abstract])) OR (Osteoarthrosis Deformans[Title/Abstract])

**#6 #4 OR #5**

**#7 #3 AND #6**

**#8** "Arthroplasty, Replacement"[Mesh]

**#9**

((((((((((((((((((Arthroplasties, Replacement[Title/Abstract]) OR (Joint Prosthesis Implantation[Title/Abstract])) OR (Implantation, Joint Prosthesis[Title/Abstract])) OR (Implantations, Joint Prosthesis[Title/Abstract])) OR (Joint Prosthesis Implantations[Title/Abstract])) OR (Prosthesis Implantation, Joint[Title/Abstract])) OR (Prosthesis Implantations, Joint[Title/Abstract])) OR (Replacement Arthroplasty[Title/Abstract])) OR (Joint Replacement[Title/Abstract])) OR (Joint Replacements[Title/Abstract])) OR (Replacement, Joint[Title/Abstract])) OR (Replacements, Joint[Title/Abstract])) OR (Replacement Arthroplasties[Title/Abstract])) OR (Total Joint Replacement[Title/Abstract])) OR (Joint Replacement, Total[Title/Abstract])) OR (Joint Replacements, Total[Title/Abstract])) OR (Replacement, Total Joint[Title/Abstract])) OR (Replacements, Total Joint[Title/Abstract])) OR (Total Joint Replacements[Title/Abstract])

**#10 #8 OR #9**

**#11 #3 AND #10**

**#12** "Arthroplasty, Replacement, Knee"[Mesh]

**#13**

((((((((((((((((((((((((((((((Arthroplasties, Replacement, Knee[Title/Abstract]) OR (Arthroplasty, Knee Replacement[Title/Abstract])) OR (Knee Replacement Arthroplasties[Title/Abstract])) OR (Knee Replacement Arthroplasty[Title/Abstract])) OR (Replacement Arthroplasties, Knee[Title/Abstract])) OR (Knee Arthroplasty, Total[Title/Abstract])) OR (Arthroplasty, Total Knee[Title/Abstract])) OR (Total Knee Arthroplasty[Title/Abstract])) OR (Replacement, Total Knee[Title/Abstract])) OR (Total Knee Replacement[Title/Abstract])) OR (Knee Replacement, Total[Title/Abstract])) OR (Knee Arthroplasty[Title/Abstract])) OR (Arthroplasty, Knee[Title/Abstract])) OR (Arhroplasties, Knee Replacement[Title/Abstract])) OR (Replacement Arthroplasty, Knee[Title/Abstract])) OR (Arthroplasty, Replacement, Partial Knee[Title/Abstract])) OR (Unicompartmental Knee Arthroplasty[Title/Abstract])) OR (Arthroplasty, Unicompartmental Knee[Title/Abstract])) OR (Knee Arthroplasty, Unicompartmental[Title/Abstract])) OR (Unicondylar Knee Arthroplasty[Title/Abstract])) OR (Arthroplasty, Unicondylar Knee[Title/Abstract])) OR (Knee Arthroplasty, Unicondylar[Title/Abstract])) OR (Partial Knee Arthroplasty[Title/Abstract])) OR (Arthroplasty, Partial Knee[Title/Abstract])) OR (Knee Arthroplasty, Partial[Title/Abstract])) OR (Unicondylar Knee Replacement[Title/Abstract])) OR (Knee Replacement, Unicondylar[Title/Abstract])) OR (Partial Knee Replacement[Title/Abstract])) OR (Knee Replacement, Partial[Title/Abstract])) OR (Unicompartmental Knee Replacement[Title/Abstract])) OR (Knee Replacement, Unicompartmental[Title/Abstract])

**#14 #12 OR #13**

**#15 #7 AND #11 AND #14**

**Embase**

**#1** ‘'osteoarthritis'/exp

**#2**

'osteoarthritides':ab,ti OR 'osteoarthrosis':ab,ti OR 'osteoarthroses':ab,ti OR 'arthritis, degenerative':ab,ti OR 'arthritides, degenerative':ab,ti OR 'degenerative arthritides':ab,ti OR 'degenerative arthritis':ab,ti OR 'arthrosis':ab,ti OR 'arthroses':ab,ti

**#3: #1 OR #2**

**#4** 'patellofemoral joint'/exp

**#5**

'joint, patellofemoral':ab,ti OR 'joints, patellofemoral':ab,ti OR 'patellofemoral joints':ab,ti OR 'patellofemoral articulation':ab,ti OR 'articulation, patellofemoral':ab,ti OR 'articulations, patellofemoral':ab,ti OR 'patellofemoral articulations':ab,ti OR 'femoropatellar articulation':ab,ti OR 'articulation, femoropatellar':ab,ti OR 'articulations, femoropatellar':ab,ti OR 'femoropatellar articulations':ab,ti

**#6: #4 OR #5**

**#7 #3 AND #6**

**#8** 'replacement arthroplasty'/exp

**#9**

'arthroplasties, replacement':ab,ti OR 'joint prosthesis implantation':ab,ti OR 'implantation, joint prosthesis':ab,ti OR 'implantations, joint prosthesis':ab,ti OR 'joint prosthesis implantations':ab,ti OR 'prosthesis implantation, joint':ab,ti OR 'prosthesis implantations, joint':ab,ti OR 'replacement arthroplasty':ab,ti OR 'joint replacement':ab,ti OR 'joint replacements':ab,ti OR 'replacement, joint':ab,ti OR 'replacements, joint':ab,ti OR 'replacement arthroplasties':ab,ti OR 'total joint replacement':ab,ti OR 'joint replacement, total':ab,ti OR 'joint replacements, total':ab,ti OR 'replacement, total joint':ab,ti OR 'replacements, total joint':ab,ti OR 'total joint replacements':ab,ti

**#10: #8 OR #9**

**#11:#6 AND #10**

**#12** 'knee replacement'/exp

**#13**

'arthroplasties, replacement, knee':ab,ti OR 'arthroplasty, knee replacement':ab,ti OR 'knee replacement arthroplasties':ab,ti OR 'knee replacement arthroplasty':ab,ti OR 'replacement arthroplasties, knee':ab,ti OR 'knee arthroplasty, total':ab,ti OR 'arthroplasty, total knee':ab,ti OR 'total knee arthroplasty':ab,ti OR 'replacement, total knee':ab,ti OR 'total knee replacement':ab,ti OR 'knee replacement, total':ab,ti OR 'knee arthroplasty':ab,ti OR 'arthroplasty, knee':ab,ti OR 'arhroplasties, knee replacement':ab,ti OR 'replacement arthroplasty, knee':ab,ti OR 'arthroplasty, replacement, partial knee':ab,ti OR 'unicompartmental knee arthroplasty':ab,ti OR 'arthroplasty, unicompartmental knee':ab,ti OR 'knee arthroplasty, unicompartmental':ab,ti OR 'unicondylar knee arthroplasty':ab,ti OR 'arthroplasty, unicondylar knee':ab,ti OR 'knee arthroplasty, unicondylar':ab,ti OR 'partial knee arthroplasty':ab,ti OR 'arthroplasty, partial knee':ab,ti OR 'knee arthroplasty, partial':ab,ti OR 'unicondylar knee replacement':ab,ti OR 'knee replacement, unicondylar':ab,ti OR 'partial knee replacement':ab,ti OR 'knee replacement, partial':ab,ti OR 'unicompartmental knee replacement':ab,ti OR 'knee replacement, unicompartmental':ab,ti

**#14 #12 OR #13**

**#15 #7 AND #11 AND #14**

**Cochrane**

**#1**

MeSH descriptor: [Osteoarthritis] explode all trees

**#2**

(Osteoarthritides):ti,ab,kw OR (Osteoarthrosis):ti,ab,kw OR (Osteoarthroses):ti,ab,kw OR (Arthritis, Degenerative):ti,ab,kw OR (Arthritides, Degenerative):ti,ab,kw OR (Degenerative Arthritides):ti,ab,kw OR (Degenerative Arthritis):ti,ab,kw OR (Arthrosis):ti,ab,kw OR (Arthroses):ti,ab,kw OR (Osteoarthrosis Deformans):ti,ab,kw

**#3: #1 OR #2**

**#4**

MeSH descriptor: [Patellofemoral Joint] explode all trees

**#5**

(Joint, Patellofemoral):ti,ab,kw OR (Joints, Patellofemoral):ti,ab,kw OR (Patellofemoral Joints):ti,ab,kw OR (Patellofemoral Articulation):ti,ab,kw OR (Articulation, Patellofemoral):ti,ab,kw OR (Articulations, Patellofemoral):ti,ab,kw OR (Patellofemoral Articulations):ti,ab,kw OR (Femoropatellar Articulation):ti,ab,kw OR (Articulation, Femoropatellar):ti,ab,kw OR (Articulations, Femoropatellar):ti,ab,kw OR (Femoropatellar Articulations):ti,ab,kw

**#6: #4 OR #5**

**#7 #3 AND #6**

**#8**

MeSH descriptor: [Arthroplasty, Replacement] explode all trees

**#9**

(Arthroplasties, Replacement):ti,ab,kw OR (Joint Prosthesis Implantation):ti,ab,kw OR (Implantation, Joint Prosthesis):ti,ab,kw OR (Implantations, Joint Prosthesis):ti,ab,kw OR (Joint Prosthesis Implantations):ti,ab,kw OR (Prosthesis Implantation, Joint):ti,ab,kw OR (Prosthesis Implantations, Joint):ti,ab,kw OR (Replacement Arthroplasty):ti,ab,kw OR (Joint Replacement):ti,ab,kw OR (Joint Replacements):ti,ab,kw OR (Replacement, Joint):ti,ab,kw OR (Replacements, Joint):ti,ab,kw OR (Replacement Arthroplasties):ti,ab,kw OR (Total Joint Replacement):ti,ab,kw OR (Joint Replacement, Total):ti,ab,kw OR (Joint Replacements, Total):ti,ab,kw OR (Replacement, Total Joint):ti,ab,kw OR (Replacements, Total Joint):ti,ab,kw OR (Total Joint Replacements):ti,ab,kw

**#10: # 8 OR #9**

**#11: #6 AND 10**

**#12**

MeSH descriptor: [Arthroplasty, Replacement, Knee] explode all trees

**#13**

(Arthroplasties, Replacement, Knee):ti,ab,kw OR (Arthroplasty, Knee Replacement):ti,ab,kw OR (Knee Replacement Arthroplasties):ti,ab,kw OR (Knee Replacement Arthroplasty):ti,ab,kw OR (Replacement Arthroplasties, Knee):ti,ab,kw OR (Knee Arthroplasty, Total):ti,ab,kw OR (Arthroplasty, Total Knee):ti,ab,kw OR (Total Knee Arthroplasty):ti,ab,kw OR (Replacement, Total Knee):ti,ab,kw OR (Total Knee Replacement):ti,ab,kw OR (Knee Replacement, Total):ti,ab,kw OR (Knee Arthroplasty):ti,ab,kw OR (Arthroplasty, Knee):ti,ab,kw OR (Arhroplasties, Knee Replacement):ti,ab,kw OR (Replacement Arthroplasty, Knee):ti,ab,kw OR (Arthroplasty, Replacement, Partial Knee):ti,ab,kw OR (Unicompartmental Knee Arthroplasty):ti,ab,kw OR (Arthroplasty, Unicompartmental Knee):ti,ab,kw OR (Knee Arthroplasty, Unicompartmental):ti,ab,kw OR (Unicondylar Knee Arthroplasty):ti,ab,kw OR (Arthroplasty, Unicondylar Knee):ti,ab,kw OR (Knee Arthroplasty, Unicondylar):ti,ab,kw OR (Partial Knee Arthroplasty):ti,ab,kw OR (Arthroplasty, Partial Knee):ti,ab,kw OR (Knee Arthroplasty, Partial):ti,ab,kw OR (Unicondylar Knee Replacement):ti,ab,kw OR (Knee Replacement, Unicondylar):ti,ab,kw OR (Partial Knee Replacement):ti,ab,kw OR (Knee Replacement, Partial):ti,ab,kw OR (Unicompartmental Knee Replacement):ti,ab,kw OR (Knee Replacement, Unicompartmental):ti,ab,kw

**#14 #12 OR #13**

**#15 #7 AND #11 AND #14**

**Web of Science**

**#1**

TS=(Osteoarthritis or Osteoarthritides or Osteoarthrosis or Osteoarthroses or Arthritis, Degenerative or Arthritides, Degenerative or Degenerative Arthritides or Degenerative Arthritis or Arthrosis or Arthroses or Osteoarthrosis Deformans )

**#2**

TS=(Patellofemoral Joint or Joint, Patellofemoral or Joints, Patellofemoral or Patellofemoral Joints or Patellofemoral Articulation or Articulation, Patellofemoral or Articulations, Patellofemoral or Patellofemoral Articulations or Femoropatellar Articulation or Articulation, Femoropatellar or Articulations, Femoropatellar or Femoropatellar Articulations)

**#3 #2 AND #1**

**#4**

TS=(Arthroplasty, Replacement or Arthroplasties, Replacement or Joint Prosthesis Implantation or Implantation, Joint Prosthesis or Implantations, Joint Prosthesis or Joint Prosthesis Implantations or Prosthesis Implantation, Joint or Prosthesis Implantations, Joint or Replacement Arthroplasty or Joint Replacement or Joint Replacements or Replacement, Joint or Replacement, Joint or Replacement, Joint or Replacement, Joint or Replacement, Joint or Replacement, Joint or Replacement, Joint or Replacement, Joint or Replacement, Joint)

**#5 #4 AND #2**

**#6**

TS=(Arthroplasty, Replacement, Knee or Arthroplasties, Replacement, Knee or Arthroplasty, Knee Replacement or Knee Replacement Arthroplasties or Knee Replacement Arthroplasty or Replacement Arthroplasties, Knee or Knee Arthroplasty, Total or Arthroplasty, Total Knee or Total Knee Arthroplasty or Replacement, Total Knee or Total Knee Replacement or Knee Replacement, Total or Knee Arthroplasty or Arthroplasty, Knee or Arhroplasties, Knee Replacement or Replacement Arthroplasty, Knee or Arthroplasty, Replacement, Partial Knee or Unicompartmental Knee Arthroplasty or Arthroplasty, Unicompartmental Knee or Knee Arthroplasty, Unicompartmental or Unicondylar Knee Arthroplasty or Arthroplasty, Unicondylar Knee or Knee Arthroplasty, Unicondylar or Partial Knee Arthroplasty or Arthroplasty, Partial Knee or Knee Arthroplasty, Partial or Unicondylar Knee Replacement or Knee Replacement, Unicondylar or Partial Knee Replacement or Knee Replacement, Partial or Unicompartmental Knee Replacement or Knee Replacement, Unicompartmental)

**#7 #6 AND #5 AND #3**
