# Supplementary material for: Patellofemoral arthroplasty versus total knee arthroplasty for isolated patellofemoral osteoarthritis: a systematic review and meta-analysis
Source: J Orthop Surg Res. 2021 Apr 15;16:264. doi: 10.1186/s13018-021-02414-5 (PMC8048312; doi:10.1186/s13018-021-02414-5)
Supplement: Supplementary file 2 — Additional file 2. Methodological assessment according to seven domains of potential biases (ROBINS-I). [file 13018_2021_2414_MOESM2_ESM.docx]

**Appendix 2** Methodological assessment according to seven domains of potential biases (ROBINS-I)

| nRCT  Study=4 | Bias due to  confounding | Bias in selection of  participants | Bias in measurement  of interventions | Bias due to departures from intended interventions | Bias due to missing data | Bias in measurement  of out comes | Bias in selection of the reported result | Overall bias |
| --- | --- | --- | --- | --- | --- | --- | --- | --- |
| Clement et al. [25] (2019) | moderate | low | low | low | moderate | moderate | moderate | moderate |
| Kamikovski et al. [28] (2019) | low | moderate | low | low | moderate | moderate | moderate | moderate |
| Perrone et al.  [29] (2018) | moderate | Serious | low | low | low | moderate | moderate | Serious |
| Dahm et al.  [26] (2010) | low | moderate | low | low | low | low | moderate | moderate |

nRCT: Nonrandomized controlled trial; ROBINS-I: Risk Of Bias In Non-randomized Studies of Interventions.
