# Supplementary material for: Patellofemoral arthroplasty versus total knee arthroplasty for isolated patellofemoral osteoarthritis: a systematic review and meta-analysis
Source: J Orthop Surg Res. 2021 Apr 15;16:264. doi: 10.1186/s13018-021-02414-5 (PMC8048312; doi:10.1186/s13018-021-02414-5)
Supplement: Supplementary file 3 — Additional file 3. Methodological assessment according to six domains of potential biases(Cochrane Risk of Bias Tool). [file 13018_2021_2414_MOESM3_ESM.docx]

**Appendix 3** Methodological assessment according to six domains of potential biases(Cochrane Risk of Bias Tool)

| RCT  Study=3 | Random  Sequence generation | Allocation  concealment | Blinding of participants  and personnel | Blinding of  Outcome assessment | Incomplete  Outcome data | Selective  reporting | Other bias | Overall bias |
| --- | --- | --- | --- | --- | --- | --- | --- | --- |
| Fredborg et al. [15]  (2020) | low | unclear | unclear | unclear | high | unclear | unclear | high |
| Joseph et al. [27]  (2020) | low | low | unclear | unclear | low | unclear | unclear | low |
| Odgaard et al. [20]  (2018) | low | low | unclear | low | low | unclear | unclear | low |

RCT: Randomized controlled trial.
